# Validation of MALDI-MS imaging data of selected membrane lipids in murine brain with and without laser postionization by quantitative nano-HPLC-MS using laser microdissection

**DOI:** 10.1007/s00216-020-02818-y

**Published:** 2020-07-25

**Authors:** Fabian B. Eiersbrock, Julian M. Orthen, Jens Soltwisch

**Affiliations:** 1 Institute of Hygiene, Westfälische Wilhelms-Universität Münster, Robert-Koch-Straße 41, 48149 Münster, Germany; 2 Interdisciplinary Center for Clinical Research (IZKF), Westfälische Wilhelms-Universität Münster, Domagkstraße 3, 48149 Münster, Germany

**Keywords:** MALDI, Mass spectrometry imaging, Laser postionization, MALDI-2, Quantification, Signal intensity response, Laser microdissection, Nano-HILIC-nano-ESI-MS, Lipids

## Abstract

MALDI mass spectrometry imaging (MALDI-MSI) is a widely used technique to map the spatial distribution of molecules in sectioned tissue. The technique is based on the systematic generation and analysis of ions from small sample volumes, each representing a single pixel of the investigated sample surface. Subsequently, mass spectrometric images for any recorded ion species can be generated by displaying the signal intensity at the coordinate of origin for each of these pixels. Although easily equalized, these recorded signal intensities, however, are not necessarily a good measure for the underlying amount of analyte and care has to be taken in the interpretation of MALDI-MSI data. Physical and chemical properties that define the analyte molecules’ adjacencies in the tissue largely influence the local extraction and ionization efficiencies, possibly leading to strong variations in signal intensity response. Here, we inspect the validity of signal intensity distributions recorded from murine cerebellum as a measure for the underlying molar distributions. Based on segmentation derived from MALDI-MSI measurements, laser microdissection (LMD) was used to cut out regions of interest with a homogenous signal intensity. The molar concentration of six exemplary selected membrane lipids from different lipid classes in these tissue regions was determined using quantitative nano-HPLC-ESI-MS. Comparison of molar concentrations and signal intensity revealed strong deviations between underlying concentration and the distribution suggested by MSI data. Determined signal intensity response factors strongly depend on tissue type and lipid species.

**Electronic supplementary material:**

The online version of this article (10.1007/s00216-020-02818-y) contains supplementary material, which is available to authorized users.

**Figure:**
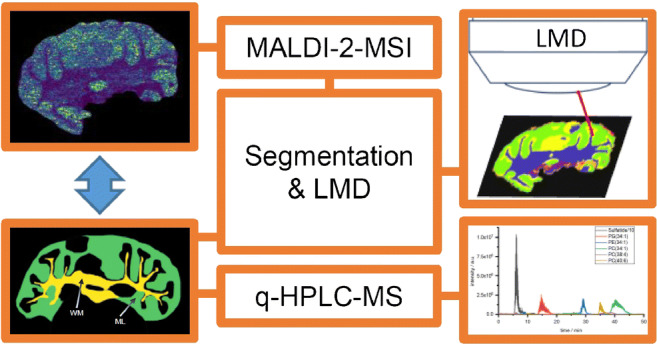
Graphical abstract

## Introduction

Combining the analytical benefits of mass spectrometric analysis with spatial information, mass spectrometry imaging (MSI) has evolved into an important analytical tool in a wide field of application ranging from quality control in process management to the analysis of tissue samples in the life sciences [1, 2]. Common to all MSI techniques, ions are produced from a defined spot or pixel on the sample surface and are analyzed by mass spectrometry. Subsequently, MS data are combined with their spatial origin in order to visualize the spatial distribution of signal intensities for each measured *m/z* value. Matrix-assisted laser desorption/ionization- mass spectrometry (MALDI-MS) represents one of the most popular methods for MSI of intact biomolecules [3]. Here, the sample is covered with a homogenous layer of a chemical matrix by spraying or sublimation/recrystallization [4, 5]. During application of this matrix, analyte molecules are extracted into the layer of matrix coating and co-crystalize within. Upon irradiation with ns-long pulses of UV laser light, the crystal lattice rapidly disintegrates, co-desorbing and ionizing imbedded analyte molecules. While the ionization process in MALDI is still under discussion, it is widely accepted that only a very small fraction of desorbed molecules is ionized [6–13]. Ion yields strongly depend on the chemical properties of the matrix and the analyte itself but also on the overall composition of the sample [12, 14, 15]. For glycerophospholipids (GPL), for example, it has been shown that the presence of small quantities of phosphatidylcholine (PC) can effectively quench ion yields for other GPL classes like phosphatidylethanolamine (PE) [15, 16]. This phenomenon is often called ion suppression effect (ISE) and can be observed for a wide variety of analyte classes [17–20]. Best viewed as a collective term for a group of effects rather than the result of a single defined mechanism, ISE may depend on physical-chemical properties of the matrix and the interplay of suppressed and suppressing analyte. Also, matrix preparation protocol as well as the “chemical background” of the sample may influence ISE [14, 21]. While in the analysis of extracted analyte upstream separation techniques like high performance liquid chromatography (HPLC) can be used to reduce sample complexity and omit ISE, MALDI-MSI inherently handles unseparated samples. In order to reduce ISE for the targeted analysis of certain heavily suppressed analyte classes, on tissue enzymatic digestion to deplete suppressing substances and on tissue derivatization to increase sensitivity for a certain analyte class have been successfully employed [22–24]. In a different approach, laser-induced postionization in a fine vacuum MALDI ion source (MALDI-2) has shown promising first results pointing towards a reduction in ISE in the analysis of membrane lipids [15, 25–27]. In this technique, a second laser pulse hits the evolving plume 10 μs after the initial MALDI event ca. 500 μm above the sample surface. Induced by resonance-enhanced two-photon ionization of matrix molecules, a reaction cascade within the plume leads to an efficient postionization for a large number of lipid classes. Interestingly, lipid classes strongly suffering from ISE benefit the most from the boost in signal intensity provided by MALDI-2 in the form of additional protonated ions. In MALDI, analyte species are usually detected carrying a single charge formed by adduct formation with a proton or alkali metal cation in positive ion mode and the subtraction of a proton in negative ion mode. Especially for GPL and other membrane lipids, often detected as sodiated or potassiated species, ion formation is therefore also dependent on the local salt composition [28, 29]. Apart from ionization efficiencies, also the physical and chemical properties of the underlying tissue can influence signal intensity by affecting analyte extraction into the matrix [30–32].

GPLs and other membrane lipids are among the prime targets of MALDI-MSI. As key constituents of cellular membranes, they are ubiquitous to most biological tissue and their singly charged *m/z* values lay in a range easily accessible by most MS analyzers. Inherent to their amphipathic character, most membrane lipids comprise a hydrophilic head-group and a lipophilic tail. With at least six different head groups regularly found in the membranes of mammals for GPLs alone and their lipophilic tail varying in length of its acyl chains as well their degree and position of unsaturation, lipid composition in most tissue samples is highly complex. This complexity leads to the occurrence of isobaric species and renders a complete identification of lipid species impossible if based on accurate mass alone [33, 34]. Although further structural elucidation is possible by different tandem MS strategies coupled to MSI, often in combination with chemical derivatization [34–39], annotation of membrane lipids is therefore often reduced to the description of the head group and the total number of carbons as well as the number of C–C double bonds in the acyl chains (e.g., PC(40:6)) [40]. Next to isobaric species rooted in variations in the acyl chains, also differences in ion type can produce near-isobaric interference. A prominent example to this end can be found in [PC(40:1) + Na]^+^ and [PC(40:4) + H]^+^ or any other combination with the same difference in chain length and number of double bonds producing ion species varying by only 2.408 mDa.

In MALDI-MSI, results are most often presented as color-coded maps displaying measured signal intensity at each pixel. While it is alluring to equalize these intensity distributions of a certain *m/z* value with the underlying concentration of a certain compound putatively assigned by accurate mass, great care has to be taken interpreting MSI data. Signal intensity for a certain *m/z* value recorded at a certain pixel is not solely determined by the underlying content of a single molecular species but influenced by parameters like the ones described above. These effects may change drastically at different areas of the investigated sample causing misleading artifacts in the visualization [32, 41]. Uncorrected MS images may therefore not depict the spatial distribution of a specific molecular species but are often distorted by secondary factors, greatly hampering their quantitative but also qualitative interpretation. To address this issue, different solutions for quantification have been proposed [42, 43]. In a targeted approach, different research groups have used isotopically labeled standards that are homogenously deposited on top or underneath the tissue section [44–47]. Because targeted analyte as well as matched standard experience the same micro-conditions at each pixel, signal intensity from the standard can be used for normalization. Extraction from the tissue however is in this case not ideally reproduced for the standard. In another targeted approach for the analysis of exogenous analytes, different researchers introduced tissue-type-specific extraction coefficients [48–50]. This was determined by producing homogenates of each subtype of tissue spiked with different concentrations of the target analyte. Cryosections of these homogenates were analyzed to determine tissue extraction coefficients. While laborious, both approaches allowed for quantitative MALDI-MSI of the respective target substance. For untargeted MALDI-MSI of endogenous compounds like GPLs, however, reliable quantification strategies are still largely missing.

In order to identify and assess the severity of possible pitfalls in the interpretation of signal intensity maps of GPLs, we here present a workflow that allows quantifying lipid content in tissue areas as identified by MALDI-MSI. By using laser microdissection (LMD) on the same sample after MALDI-MSI analysis similar to published work flows for proteins and lipids [51–54], lipid extracts were produced for selected areas and analyzed by quantitative nano-HPLC-nano-ESI-MS. Comparison of quantitative data and MS images allowed assessing the value of the signal intensity maps from a quantitative point of view. A total of six lipid species from different GPL classes (PC(34:1), PC(38:4), PC(40:6), PE(34:1), phosphatidylglycerol(34:1) (PG(34:1))) and one sulfatide ((3′-sulfo)Galβ1-1Cer(d18:1/24:1)) were analyzed. White matter of the arbor vitae and gray matter of the molecular layer from murine cerebellum were chosen as representative tissue regions.

## Materials and methods

### Chemicals and reagents

All solvents were purchased from Carl Roth (Karlsruhe, Germany). All other chemicals were purchase from Sigma-Aldrich (Merck, Darmstadt, Germany), if not declared otherwise.

### Workflow

**Fig. 1:**
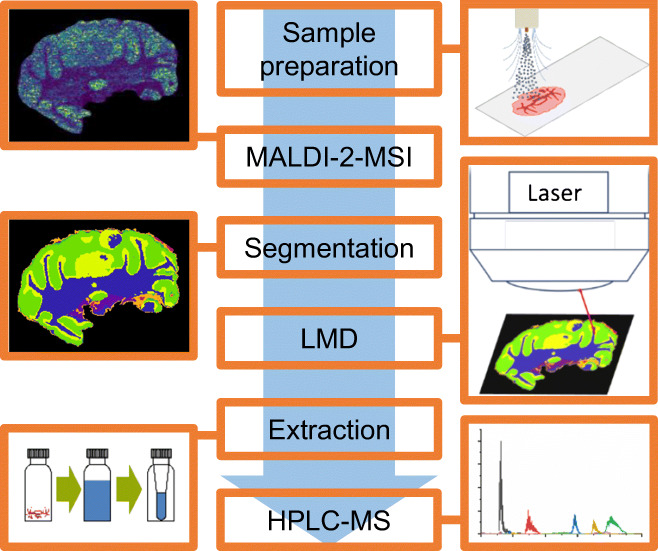
Schematic workflow for comparative MALDI and MALDI-2-MSI and quantitative HPLC-ESI-MS

Figure 1 depicts a schematic general workflow used for comparative MS imaging and quantitative HPLC-MS analysis. After sample preparation and MALDI-MSI analysis, regions of interest (ROI) are identified based on MSI results and dissected using LMD. Lipids from dissected tissue regions are extracted and analyzed using quantitative nano-HILIC HPLC-ESI-MS.

### Sample preparation for MALDI-MSI

Mouse brain samples originated from 11- to 13-week-old female C57BI/6 mice. The brains were extracted and flash frozen in liquid nitrogen directly after sacrifice and stored at − 80 °C until use. Cryosectioning at − 20 °C was performed using a Jung Frigocut 2800-E cryotome (Leica, Wetzlar, Germany). The sections were transferred via thaw mounting to common microscopy glass slides (Superfrost, Thermo Fisher Scientific, Darmstadt, Germany) or special membrane slides with a polyethylene naphthalate (PEN) membrane for later LMD (P.A.L.M. Microlaser Technologies, Bernried, Germany). All sections in this study are consecutive cuts from the same mouse brain with 8 μm thickness for H&E staining and 20 μm for MALDI-MSI and later HPLC-MS experiments. 2,5-Dihydroxyacetophenone (DHAP) was used as a MALDI matrix. A total of 20 mg/mL of DHAP was dissolved in acetonitrile/water/methanol (75/20/5; v/v/v) with 0.1% formic acid for later spray coating of the sample. Matrix solution was sprayed onto the tissue with an ultrasonic sprayer (SimCoat with AccuMist nozzle, Sono-Tek, Milton, NY, USA). The sprayer was operated in serpentine mode. One cycle was made up of respective sets of horizontal and vertical passes. Line spacing was 1.8 mm and velocity was 30 mm/s. Flow rate of the solution was 0.05 mL/min. Matrix application was finished after ten cycles.

### MALDI-MSI measurements

A Q Exactive plus Orbitrap Mass Spectrometer (Thermo Fisher Scientific, Bremen, Germany) was used for all measurements (MALDI-MSI, MALDI-2-MSI, and HPLC-nESI-MS). The usual MS inlet was replaced with a MALDI/ESI injector (Spectroglyph LLC, Kennewick, WA, USA) to perform MALDI and ESI experiments on the same instrument [55]. The injector was modified to enable laser postionization (MALDI-2) as described before [35]. A frequency-tripled actively Q-switched Nd:YLF laser (Explorer ICT-349-120-E, Spectra-Physics; emission wavelength = 349 nm, pulse width = 7 ns; pulse repetition rate adjustable from 1 Hz to 5 kHz) was used as the MALDI laser. For postionization, a frequency-quadrupled, mode-locked Nd:YAG laser (PL2231-100-SH-FHPRETRIG, Ekspla, Vilnius, Lithuania) with a wavelength of 266 nm and a pulse duration of 28 ps was used. The MALDI laser was focused to produce ablation marks of 10–15 μm. Three consecutive sections intended for subsequent LMD and extraction were each measured with 30 μm pixel size and a mass spectrometric resolution of 70,000 at *m/z* 200 in positive ion mode. To ensure optimal image quality, MALDI as well as MALDI-2-MSI data intended for subsequent comparative analysis were collected with 20 μm pixel size and a mass spectrometric resolution of 280,000 at *m/z* 200. For this, two subsequent sections to the ones used for LMD mounted on glass slides were analyzed in positive and in negative mode, respectively. Raw data was processed to the vendor neutral .imzML format with Image Insight (Spectroglyph LLC) and further analyzed using SCiLS Lab (SCiLS, Bremen, Germany). Segmentation was performed with weak denoising. For mass spectra, refer to Figs. S1 and S2 of the Electronic Supplementary Material (ESM).

### Laser microdissection

LMD was performed on an Axiovert 200 M inverse microscope (Zeiss, Oberkochen, Germany) equipped with a LMD unit (P.A.L.M. Microlaser Technologies). PALM Robo (P.A.L.M. Microlaser Technologies) software was used to operate the system. ROIs were identified with the SCiLS Lab segmentation tool, which merges pixel with similar mass spectra. Regional coordinates were scaled and transferred to the PALM Robo software. For details, see supplementary methods in the ESM. LMD was performed without washing steps and with the matrix layer intact. After LMD, released regions of PEN membrane with attached tissue and matrix layer were mechanically transferred into a HPLC vial for lipid extraction.

### Lipid extraction and HPLC quantification

LMD, lipid extraction, and quantification were performed independently on three consecutive tissue sections. Each extract was measured in three technical replicates. The SPLASH® LIPIDOMIX® Mass Spec Standard (Avanti Polar Lipids, Alabaster, AL, USA) and (3′-sulfo)Galβ1-1Cer(d18:1/18:0*d*_3_) (Matreya, State College, PA, USA) were used as internal standards (IS) for HPLC quantification both prepared in separate solutions. Quantification was done by external calibration with internal standard. PC(16:0/18:1), PC(18:0/20:4), PC(18:0/22:6), PE(16:0/18:1), PG(16:0/18:1) (Avanti Polar Lipids), and (3′-sulfo)Galβ1-1Cer(d18:1/24:1) (Matreya) were diluted and combined to a stock solution with 1 × 10^−4^ mol/L of each compound. Nine lipid concentrations were used to construct the calibration curve. Respective calibration solutions were directly diluted from the stock (see ESM Table S1). Calibration mixtures were constructed by evaporating 5 μL solution of each IS in a glass vial and dissolving it in 20 μL of the respective calibration solution. Concentrations with high deviations in triple determination were excluded (see ESM Table S2). Before the extraction of lipids, 5 μL of each IS solution was added to the pieces of tissue regions produced by LMD. A mixture of methyl *tert*-butyl ether and methanol (MTBE/MeOH; 75/25; v/v) was used as an extracting agent for a solid/liquid extraction [56]. The tissue was extracted with 300 μL of the extracting agent for 15 min in an ultrasonic bath. The supernatant was filtered using syringe filters (0.2 μm PTFE membrane, VWR, Darmstadt, Germany). This step was repeated 2 times. The resulting extract was evaporated to dryness. To transfer the dried extract to HPLC vial micro inserts, it was vortexed with 100 μL of extraction agent three times. The transferred extract was again evaporated to dryness, dissolved in 20 μL of chloroform, and stored at − 20 °C until further use.

HPLC separation was done with an Ultimate 3000 nano-HPLC system (Thermo Fisher Scientific) and a HILIC Diol column (3 μm, 150 mm × 75 μm; Fortis Technologies, Neston, UK). Eluent A was acetonitrile (ACN). Eluent B was an ammonium acetate buffer (60 mM) in water/ACN (90/10; v/v) set to pH 4 with acetic acid. The gradient is shown in Table 1. For HPLC-MS coupling, a home build nano-ESI source, employing PicoTip™ emitters (coated silica, 15 ± 1 μm tip; New Objective, Woburn, MA, USA), was mounted to the MALDI/ESI injector. Xcalibur (Thermo Fisher Scientific) and Chromeleon software (Thermo Fisher Scientific) were used to control the Orbitrap and HPLC. HPLC-MS measurements were performed in negative ion mode with a resolution of 70,000 in a mass range from *m/z* 500 to 1500. Ionization voltage was set to − 2 kV. Pierce Negative Ion Calibration Solution (Thermo Fisher Scientific) was used for mass calibration. AGC target was set to 1E6 and maximum inlet time to 200 ms. Data evaluation was done with Xcalibur and Excel (Microsoft, Redmond, WA, USA). Peak chromatographic areas of the deprotonated ion species for the targeted sulfatide, PE, and PG and the acetate adduct for PC as well as their respective IS were evaluated for quantification.

**Table 1:** Composition and gradient of eluent used in nano-HPLC-ESI-MS

*t*/min	B/%
− 30	5
2	5
27	30
35	100
49	100
59	5

Absolute amounts of lipid as determined by quantitative HPLC were normalized to the volume of their tissue region of origin. Volume was calculated by multiplying the section thickness of 20 μm of fresh frozen tissue with the values for respective surface areas as generated by the software of the LMD system. For clarity, lipid concentrations in solution are described as mol/L while concentrations in fresh frozen tissue are presented with the unit nmol/mm^3^ throughout the manuscript. For an exemplary chromatogram, mass spectra and calibration curves refer to Figs. S3 and S4 of the ESM.

### Hematoxylin and eosin stain

Mayer’s hemalum solution (Merck, Darmstadt, Germany) was applied for 1 min and rinsed of with tap water. Eosin Y solution (0.5%, aqueous; Merck, Darmstadt, Germany) was applied for 1 min and briefly rinsed with tap water. After drying, the slides were observed with the microscope and scanned with a slide scanner (reflecta, Eutingen, Germany).

## Results

### MALDI-imaging

**Fig. 2:**
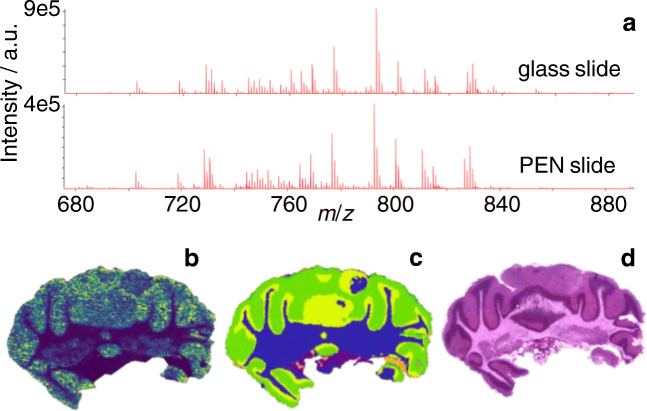
MALDI-2-MSI of mouse cerebellum. **a** Comparison of average spectra acquired from comparable tissue sections of mouse cerebellum mounted on regular glass slide (top) and PEN slide material (bottom) in positive ion mode. **b** Exemplary MALDI-2-MS image of *m/z* 792.55 (presumably [PE(40:6) + H]^+^) recorded in undersampling mode (square pixel size 30 μm; round ablation mark 15 μm in diameter). **c** Result of segmentation performed with SCiLS lab software. **d** Optical image of H&E-stained section (located 40 μm behind section used in **b**)

**Fig. 3:**
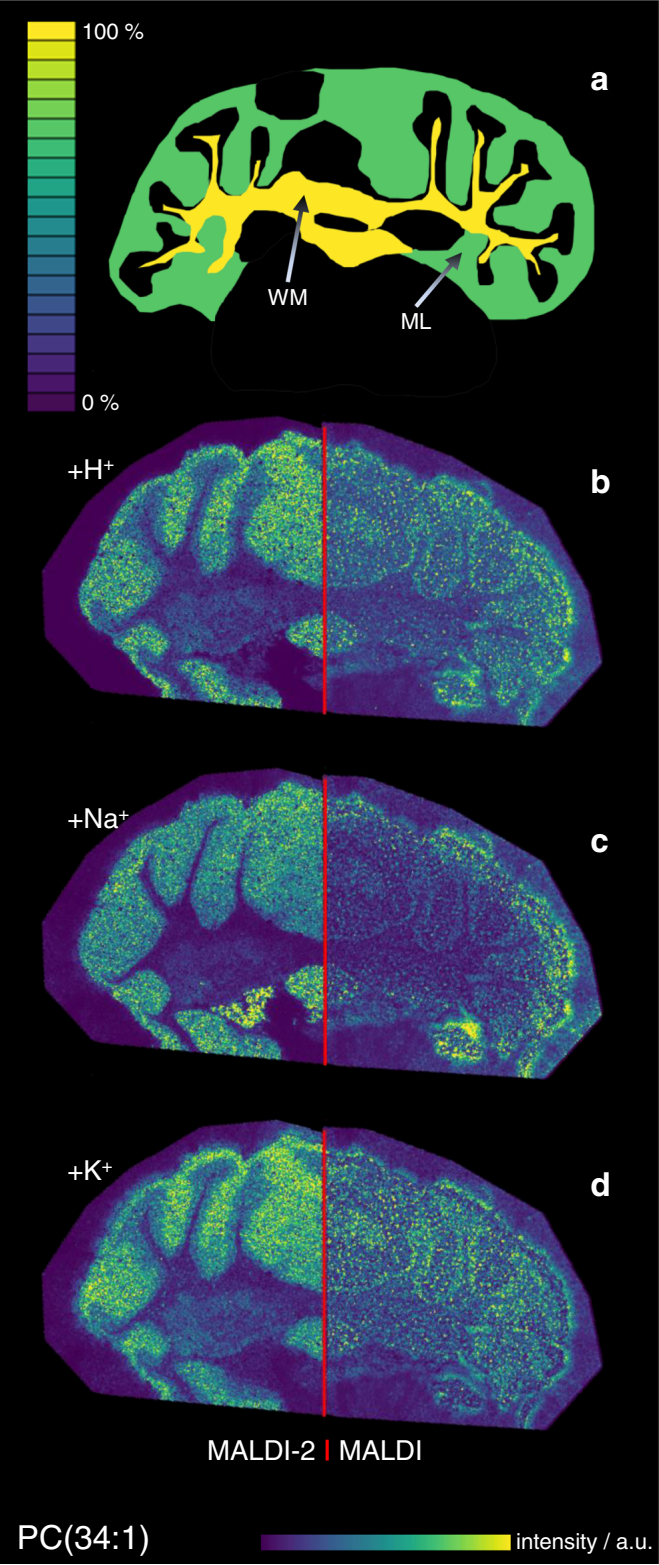
Comparison of positive ion mode MALDI and MALDI-2-MSI signal intensity maps with underlying molecular content on the example of PC(34:1). **a** Schematic depiction of the molar distribution of PC(34:1) in the white matter (WM) and molecular layer (ML) based on quantitative nano-HPLC-ESI-MS analysis after laser microdissection and solid-liquid extraction. **b**–**d** Signal intensity distribution of [PC(34:1) + H]^+^ (**b**), [PC(34:1) + Na]^+^ (**c**), and [PC(34:1) + K]^+^ (**d**) measured from glass slide with DHAP as a matrix in positive ion mode at 20 μm pixel size using conventional MALDI (right) and MALDI-2 (left)

**Fig. 4:**
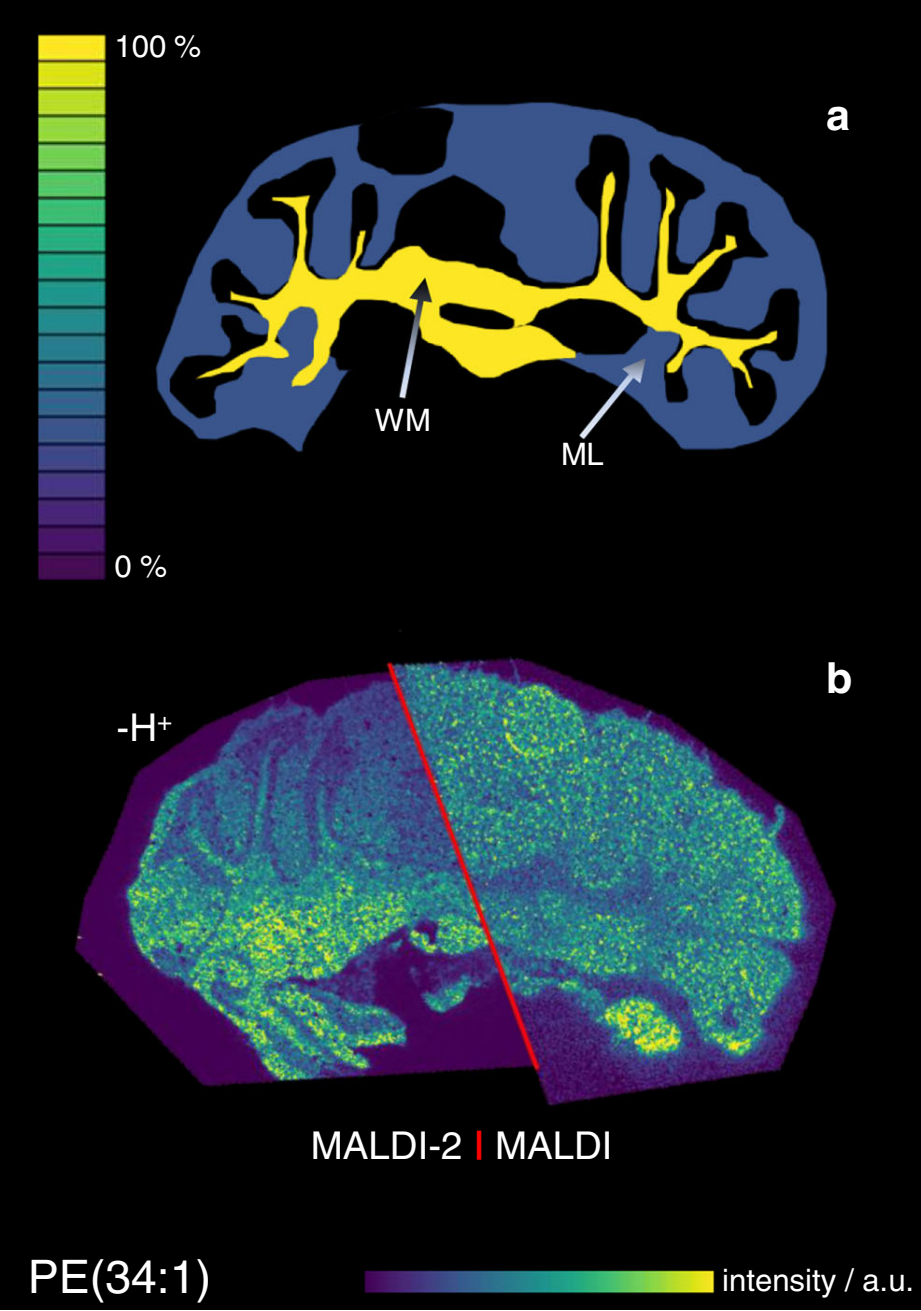
Comparison of negative ion mode MALDI and MALDI-2-MSI signal intensity maps with underlying molecular content on the example of PE(34:1). **a** Schematic depiction of the molar distribution of PE(34:1) in the white matter (WM) and molecular layer (ML) based on quantitative nano-HPLC-ESI-MS analysis after laser microdissection and solid-liquid extraction. **b** Signal intensity distribution of [PE(34:1)-H]^−^ measured from glass slide with DHAP as a matrix in negative ion mode at 20 μm pixel size using conventional MALDI (right) and MALDI-2 (left)

MALDI-2-MSI measurements dedicated for subsequent LMD and lipid extraction were performed with undersampling conditions, using a pixel size of 30 μm and an ablated area of only about 15 μm in diameter. While limiting the total amount of lipids removed or altered by laser irradiation, this allowed for the recording of MS images with reasonable mass spectrometric sensitivity and spatial resolution and enabled a subsequent segmentation of the imaging data using SCiLS (Fig. 2b and c). Next to measurements performed with undersampling on PEN slides, consecutive sections mounted on regular glass slides were investigated using MALDI and MALDI-2 with a pixel size of 20 μm and an ablated area of 15 μm in diameter (Figs. 3 and 4). While the increase in pixel density allows for a more detailed representation of tissue features, comparison of the respective mass spectra revealed no artifacts caused by the underlying PEN membrane (Fig. 2a). All targeted lipid species could be identified in either one or both measured polarities as one or more ion species. As described before, protonated ion species of usually suppressed lipid classes such as [PE(34:1) + H]^+^ benefit greatly from the application of laser postionization and are detected with a significantly enhanced signal to noise (s/n) using MALDI-2 [27]. In positive mode, PG(34:1), PE(34:1), PC(34:1), PC(38:4), and PC(40:6) were detected as protonated ions, sodium adducts, and potassium adducts in MALDI and MALDI-2 with at least 5 ppm mass accuracy (see ESM Table S3 for details). The same applies to the deprotonated ions of PG(34:1) and PE(34:1) and the sulfatide in negative mode. All targeted *m/z* values were checked for isobaric interferences in a 5-ppm-wide window by analyzing the respective ion chromatograms from HILIC-separated lipids for additional peaks. Apart from possible isotopomers within the same lipid class, no sizeable interferences with a contribution of more than 5% could be identified for any targeted lipid. As stated by different authors, normalization to the total ion count (TIC) is not suitable to reduce the observed signal intensity bias [57–59] (see ESM Fig. S5 for normalized a normalized image). While image quality increases with respect to contrast and feature recognition, inhomogeneous distribution of abundant and/or highly responsive species like the sulfatides in negative ion mode dominates the TIC and leads to the introduction of an additional layer of complexity rather than a reduction thereof. Therefore, all images represent raw signal intensity data without any type of normalization.

### Lipid extraction and HPLC-nESI-MS

Comparison of MS-based segmentation results with histological features revealed by H&E stain and microscopy, displaying a good correlation of the main segments with the arbor vitae of the white matter (WM) as well as the granular layer (GL) and molecular layer (ML) of the gray matter. Because of the thin and branched structure of the GL, located in between WM and ML, LMD was not able to confidently and reproducibly dissect and extract this part of the cerebellum. Therefore, only WM and ML were chosen for further analysis (Figs. 2, 3, and 4). After addition of standard and lipid extraction, quantitative analysis of all target lipids was successful from both sample areas (Table 2). Measured lipid concentrations are comparable with absolute concentration values reported for whole rat brain; Choi et al. also reported in the table for comparison [60]. To visualize the lipid content in both areas and for better comparability, images depicting the molecular content in WM and ML were constructed in analogy to MSI data with the color scale normalized to the higher concentration (Figs. 3a and 4a).

**Table 2:** Molar concentration of different lipids in white matter and molecular layer as determined by quantitative nano-HPLC-ESI-MS and standard deviation (SD) from triple determination and comparison to values presented by Choi et al. in [60]; Sulfatide: (3′’-sulfo)Galβ1-1Cer(d18:1/24:1)

	White matter		Molecular layer		Choi et al. whole brain*
	Mean	SD	Mean	SD	
Sulfatide	10.2	1.4	0.37	0.181	n/a
PG(34:1)	0.41	0.031	0.43	0.042	0.016
PE(34:1)	1.46	0.1	0.46	0.086	0.53
PC(34:1)	9.07	0.9	7.00	1.30	10.06
PC(38:4)	1.17	0.03	1.20	0.02	2.13
PC(40:6)	1.25	0.07	2.14	0.09	1.12

All values given in nmol/mm^3^

*Homogenate from whole rat brain; original values presented as nmol/mg, volume calculated with approx. density of 1 kg/L

### Signal intensity response in MALDI-MSI in positive ion mode

**Fig. 5:**
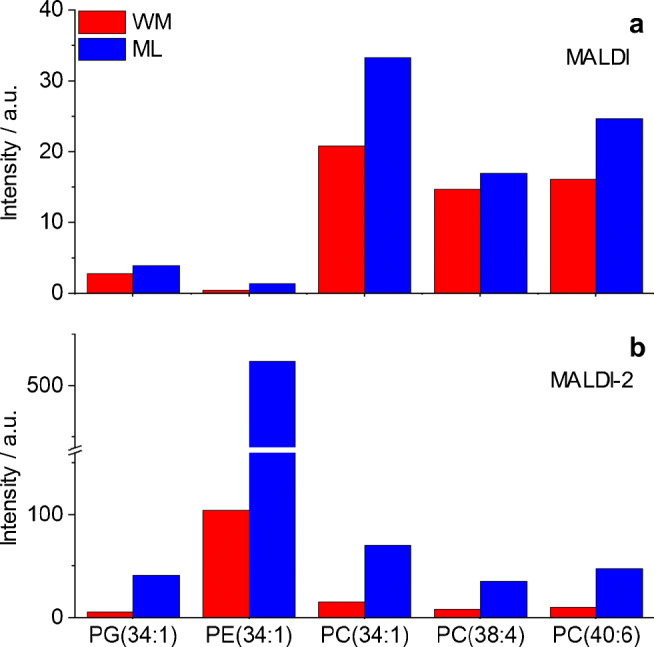
Signal intensity response from tissue in positive ion mode for different lipids. Values represent the average MALDI (**a**) or MALDI-2-MSI (**b**) signal intensity derived from the molecular concentration (nmol/mm^3^ of fresh frozen tissue) present in the tissue region for white matter (WM, red) and molecular layer (ML, blue). Average signal intensities derived from the sum of protonated, sodiated, and potassiated ion species

Figure 3 shows the signal intensity distribution for MALDI (right hemisphere) and MALDI-2 MSI (left hemisphere) compared with the underlying molar concentration in WM and ML as measured by quantitative HPLC-nESI-MS on the example of PC(34:1). Hotspot removal was applied as provided by SCiLS and signal intensity scales are normalized to the maximum value of each image. While all three ion species show similar intensity distribution in MALDI and MALDI-2, respectively, clear differences can be discerned between the two ionization modalities. Direct comparison of signal intensity distributions for MALDI as well as MALDI-2 (Fig. 3b–d) with the visualization of the underlying concentration in the tissue (Fig. 3a) reveals large discrepancies for both modalities. This can also be observed for almost all investigated lipids, where in most cases, the signal intensity distribution suggests an inverse of the actual molar distribution of the respective lipid species. To further analyze these effects, signal intensities of all accessible ion species for each target lipid in the WM and ML were summed and normalized to the size of the region by utilizing the mean intensity values provided by SCiLS for the respective ROI (ESM Tables S4 and S5). These average signal intensity values were normalized to the underlying molar concentration as revealed by the quantitative measurement. This results in a signal response value (“signal intensity per mol”) depicted in Fig. 5. For all investigated lipids, the comparison of the two regions reveals a sizably stronger response factor on ML compared with the WM. A more pronounced bias towards ML is detected in MALDI-2 compared with conventional MALDI. Comparison between the two ionization methods also reveals an increase of the response for PE and PG from both tissue types as well as PC on the ML with MALDI-2, while response from the investigated PC species from WM was largely unchanged between the two methods.

### Signal intensity response in MALDI-MSI in negative ion mode

**Fig. 6:**
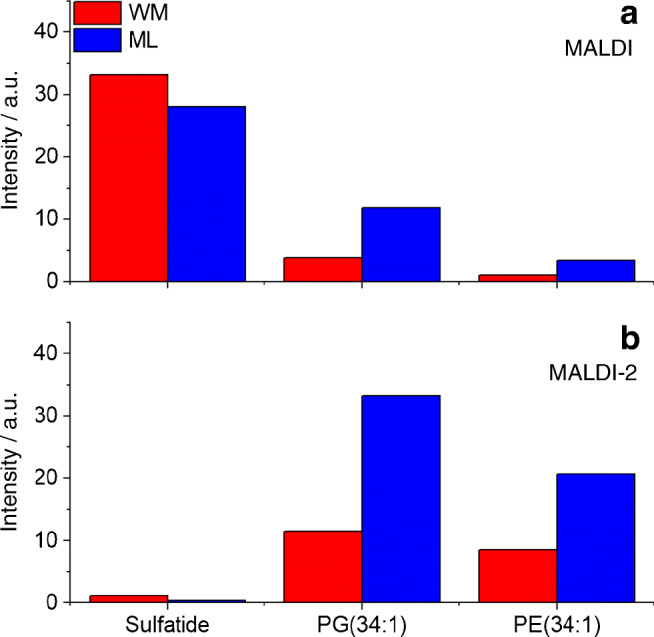
Signal intensity response from tissue in negative ion mode for different lipids. Values represent the average MALDI (**a**) or MALDI-2-MSI (**b**) signal intensity derived from the molecular concentration (nmol/mm^3^ of fresh frozen tissue) present in the tissue region for white matter (WM, red) and molecular layer (ML, blue). Average signal intensities derived from deprotonated ion species. Sulfatide: (3′-sulfo)Galβ1-1Cer(d18:1/24:1)

Analogous to the positive ion mode, experiments were also evaluated for MALDI and MALDI-2 images recorded in the negative ion mode. Here, only deprotonated lipid species of PE(34:1) and PG(34:1) and the sulfatide were detected and analyzed. Again, MALDI and MALDI-2 produce somewhat different signal intensity distributions (Fig. 4), and again discrepancies between these distributions and the underlying molar concentrations become apparent for most investigated lipid species with the exception of the sulfatide (ESM Fig. S6). In analogy to the positive ion mode, signal response factors were calculated and are displayed in Fig. 6. Similar to the positive ion mode, a strong intensity bias towards the ML over the WM is revealed for the investigated PE and PG species. In contrast, the sulfatide shows a slightly increased response from the WM. Comparing MALDI and MALDI-2, again PG and PE show an increased response with postionization for both tissue types while the response for sulfatide decreases. A similar decrease in signal intensity on peaks, dominating spectra in regular MALDI upon the use of MALDI-2 on Orbitrap instruments has been described before and is not observed in qTOF-type instruments [26].

## Discussion and conclusions

Comparative data from MALDI-MSI and quantitative HPLC-nESI-MS suggest a strong dependence of signal intensity response on the type of tissue. As described above, in both polarities and for all investigated lipid classes with the exception of sulfatide, stronger signal intensity responses were recorded from the ML compared with the WM with respect to the underlying molecular concentration. This bias could derive from a number of effects described to impact ion signal intensities in MALDI-MSI.

### Local salt concentration

In the MALDI-MSI analysis of untreated fresh frozen tissue samples in positive ion mode, sodium and potassium adducts provide the most common carrier of charge [28]. The formation of these adducts can be shifted towards one or the other, as well as other alkali metal cations by treatment with solutions of the respective acetate or protonation by the use of ammonium acetate [29]. Differences in local salt concentration between different types of tissue could, therefore, in principle induce tissue-type-specific ionization efficiencies. The effects observed here, however, are independent of the ion species. A local shift in salt concentration was therefore ruled out as the main reason for the observed bias in signal intensity response.

### Ion suppression effects

As described in the introduction, ISE are often observed in MALDI-MS of complex mixtures. In the context of membrane lipids, PC has been reported to heavily suppress all other GPL classes in the positive ion mode [16]. Recently published results by Boskamp and Soltwisch describe additional ISE between other GPL classes and also point out an inverse ion promotion effect, where the presence of a second GPL boosts ion signal intensity [15]. A variation of strongly suppressing or promoting molecular species between different types of tissue could therefore be the root cause of the observed bias in intensity response. Different indications, however, oppose the involvement of these effects. First, an ISE-induced bias would be expected to strongly influence heavily suppressed lipid classes like PE while other classes like PC should be largely unaffected. Second, ISE and promotion effects are particularly different between positive and negative ion mode [15]. As evident in Figs. 5 and 6, the bias is, however, similarly observed for different lipid classes and in both polarities. Additionally, MALDI-2 has been reported to attenuate ISE, especially for strongly suppressed lipid classes such as PE in both polarities [27]. In contrary, a stronger bias has been observed when using MALDI-2 compared with regular MALDI. For these reasons, ISE can be eliminated as the main reason for the observed effects.

### Analyte extraction and matrix morphology

Incorporation of analyte into matrix crystals or at least a close co-localization of matrix and analyte is largely accepted as an important prerequisite for a successful MALDI experiment. While in “classical” MALDI, this is usually achieved by mixing analyte and matrix in bulk solution; in MALDI-MSI, spatial distribution of analyte within the sample has to be contained during matrix application. As described above, matrix is most commonly applied by iterative spraying of matrix solution and micro-extraction within the applied droplets or, alternatively, by sublimation/recrystallization protocols. In case of spraying, the degree of extraction strongly depends on the type and amount of employed solvent as well as physical and chemical properties of the tissue [32]. For sublimation, 3-dimensional analysis of the matrix layer revealed a diffusion of lipids into the matrix layer that strongly depends on the local composition of analyte [31]. Next to extraction and incorporation of analyte, also the size and shape of matrix micro-crystals are influenced by the underlying tissue type for both types of application. Wiegelmann et al., e.g., describe strong variations in matrix morphology on WM and gray matter (GM) of mouse cerebellum [30]. In this particular case, the physical and chemical structure of WM and GM significantly differs. WM is rich in myelin that is densely folded and wrapped around nerve axons. It may therefore be speculated that extraction of membrane lipids from this tissue type into the matrix layer is significantly hampered by its structure. Boggs et al. furthermore suggest that sulfatides, among other glycolipids, abundantly decorate the outer layer of myelin bundles [61]. This may lead to an increased extraction into the matrix on WM regions and explain the distinctly different intensity response exhibited for this particular lipid. These results stand in contrast to reports on imaging techniques based on liquid extraction like nano-desorption electrospray ionization (nanoDESI) or pressurized liquid extraction surface analysis (PLESA) where no dependence of extraction efficiency on brain tissue type has been described in quantitative analysis [62, 63]. For MALDI-MSI, however, different studies have reported an influence of tissue type and preparation on the signal response for different types of lipids [18, 30, 32, 64], while others have reported a general correlation of signal intensity in MALDI-MSI with underlying molar concentrations [53, 54].

For the reasons stated above, we conclude that changes in analyte extraction and/or matrix morphology induced by the physical and chemical properties of the underlying tissue type are the main root cause for the observed bias in signal intensity response in murine brain. While factors like ion suppression and salt concentration could be counteracted by the use of external standard applied on top or underneath the tissue section, adverse effects caused by extraction from tissue can only partly be accommodated using this strategy [32, 45]. Furthermore, the use of a specific exogenous standard may limit the analysis to a small number of targeted analyte species. Omitting the use of external standard, Hankin and Murphy successfully demonstrated how normalization strategies based on an abundant endogenous PC species can be used to correct for tissue-type-specific signal response [54]. This strategy however may be limited by the availability of an abundant and homogenously distributed endogenous lipid throughout the complete tissue section and is limited to the respective class of phospholipid.

The results demonstrate that great care has to be taken in the interpretation of MALDI(-2)-MSI data and illustrate the need for robust and generally applicable quantification strategies. While laborious, MALDI-MSI-guided LMD with subsequent extraction and quantitative analysis may in the future allow to define reliable tissue-type-specific signal response factors for each class of lipids. Ultimately, this may enable a tissue-type-specific normalization without the use of exogenous standards that is guided by histological information.

## Electronic supplementary material

ESM 1 (PDF 932 kb)
