# Supplementary material for: Validation of MALDI-MS imaging data of selected membrane lipids in murine brain with and without laser postionization by quantitative nano-HPLC-MS using laser microdissection
Source: Anal Bioanal Chem. 2020 Jul 25;412(25):6875–86. doi: 10.1007/s00216-020-02818-y (PMC7496020; doi:10.1007/s00216-020-02818-y)
Supplement: Supplementary file 1 — (PDF 932 kb) [file 216_2020_2818_MOESM1_ESM.pdf]

Analytical and Bioanalytical Chemistry

Electronic Supplementary Material

Validation of MALDI-MS imaging data of selected membrane lipids in murine brain with and without laser postionization by quantitative nano-HPLC-MS using laser-microdissection

Fabian B. Eiersbrock, Julian M. Orthen, Jens Soltwisch

Table of Contents

Supplementary Methods : S3

Supplementary Figures S1-S2 : S4-S7

Supplementary Tables S1-S5 : S8-S10

Supplementary Methods

Migration of regions of interest from SCiLS Lab software to LMD instrument - To use these regions as LMD cutting patterns the size of the sample has to be determined as pixel coordinates in the PALM®Robo software. Since the ablation pattern in the matrix layer is clearly visible in the microscope, height and width can be measured with the software. The segmentation map is exported and scaled to a tenth of its final size. Scaling to the real sample size would create unreasonable big files. In a second step, the outlines of identified ROIs and of the whole measured area were marked using ImageJ 1.50i (National Institute of Health, USA), enabling an export of their border coordinates. Those coordinates were scaled up to the real sample size and imported into the PALM Robo software. The ablation pattern and the coordinates of the sample outline are now used for correct placement of the sample in the microscope.

Supplementary Figures

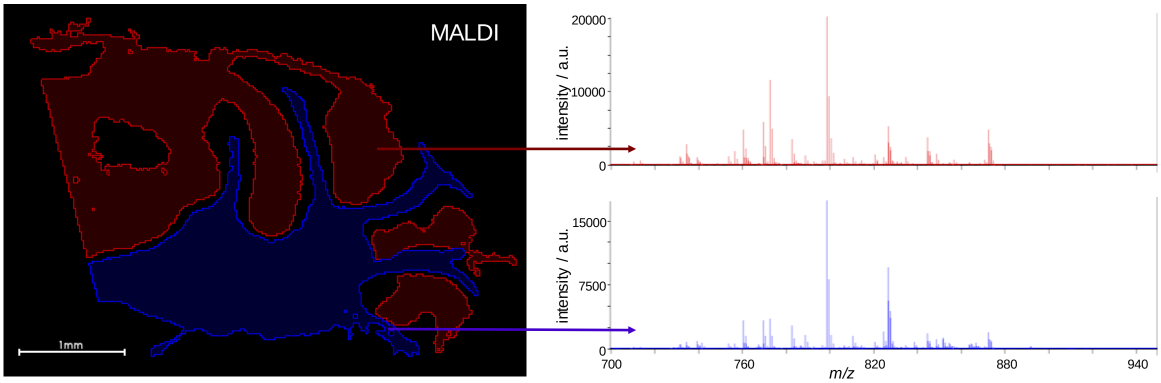

Fig. S1 Mass spectra for white matter (blue, lower panel) and molecular layer (red, upper panel) measured with MALDI-MSI in positive ion mode

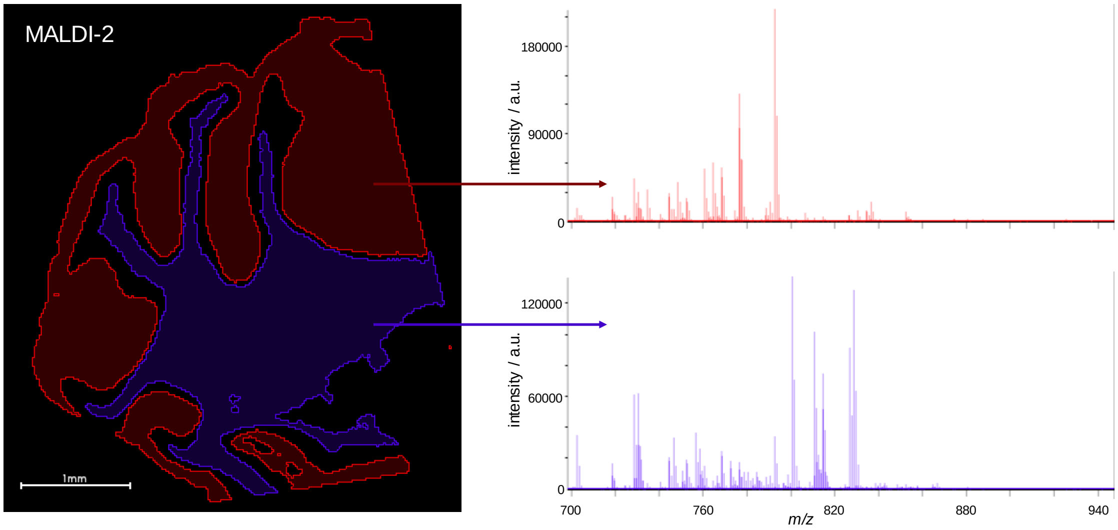

Fig. S2 Mass spectra for white matter (blue, lower panel) and molecular layer (red, upper panel) measured with MALDI-2-MSI in positive ion mode

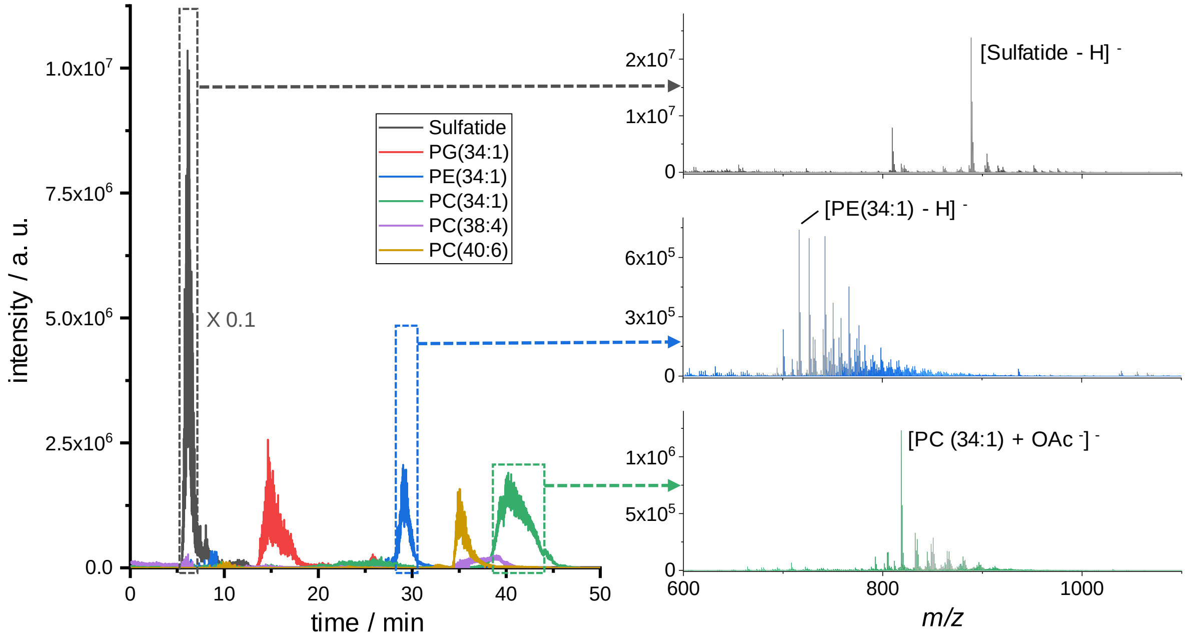

Fig. S3 Exemplary chromatogram from an extract of white matter and mass spectra of selected chromatographic peaks

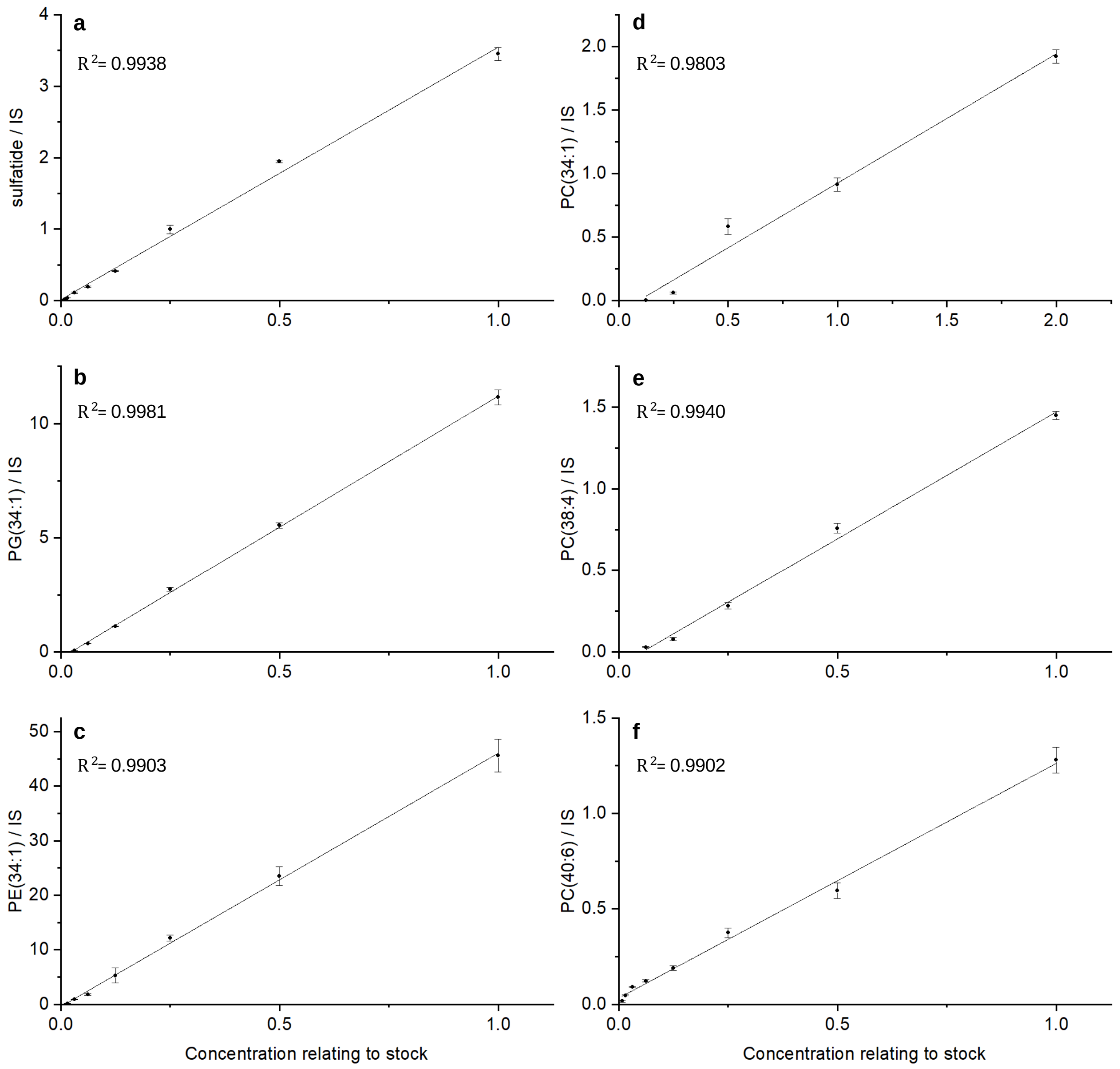

Fig. S4 Calibration curves for all targeted lipid species using the respective internal standard. For concentrations, refer to Table S1. Concentrations with high deviations in triple determination were excluded from the calibration (see Table S2)

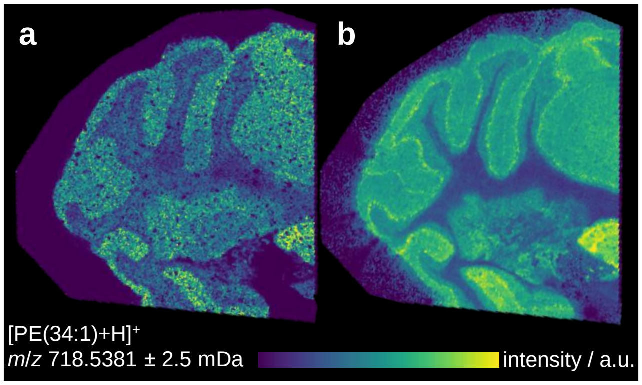

Fig. S5 Signal intensity distribution of $[PE(34:1)+H]^+$ measured with DHAP as a matrix at 20 µm pixel size and MALDI-2 in positive ion mode. Raw signal intensity data (a) and signal intensity normalized to the total ion count (TIC) in each individual pixel (b)

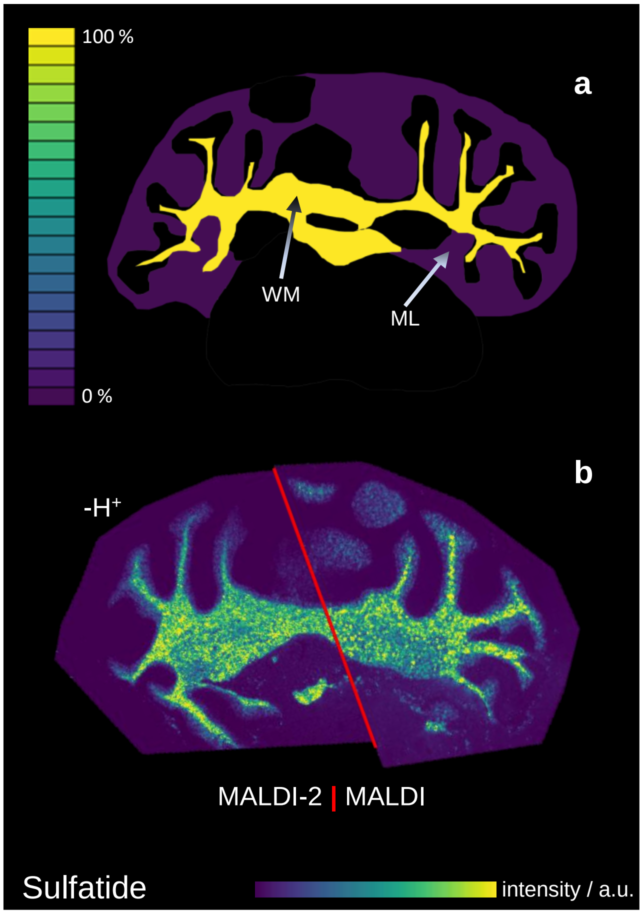

Fig. S6 Comparison of negative ion mode MALDI and MALDI-2-MSI signal intensity maps with underlying molecular content on the example of (3'-sulfo)Galβ1-1Cer(d18:1/24:1) (Sulfatide): (a) Schematic depiction of the molar distribution the white matter (WM) and molecular layer (ML) based on quantitative nanoHPLC-ESI-MS analysis after laser microdissection and solid-liquid extraction; (b) signal intensity distribution of the deprotonated ion species measured from glass slide with DHAP as a matrix in negative ion mode at 20 µm pixel size using conventional MALDI (right) and MALDI-2 (left).

Supplementary Tables

Table S1 Concentration of standards used for calibration in quantitative nano-HPLC-ESI-MS

Concentration relating to stock	Concentration / mol·L^{-1}
2	2.0000E-04
1	1.0000E-04
1/2	5.0000E-05
1/4	2.5000E-05
1/8	1.2500E-05
1/16	6.2500E-06
1/32	3.1250E-06
1/64	1.5625E-06
1/128	7.8125E-07

Table S2 Standard concentrations marked with ✓ were included in the calibration. Concentrations with high deviation in triple determination are marked with × and were excluded from calibration

	2	1	1/2	1/4	1/8	1/16	1/32	1/64	1/128
Sulfatide	×	✓	✓	✓	✓	✓	✓	✓	✓
PG (34:1)	×	✓	✓	✓	✓	✓	✓	×	×
PE (34:1)	×	✓	✓	✓	✓	✓	✓	✓	×
PC (34:1)	✓	✓	✓	✓	✓	×	×	×	×
PC (38:4)	×	✓	✓	✓	✓	✓	×	×	×
PC (40:6)	×	✓	✓	✓	✓	✓	✓	✓	✓

Table S3 Measured and calculated *m/z* values for all ion species investigated with MALDI- and MALDI-2-MSI for positive (+H, +Na, +K) and negative ion mode (-H)

	m/z (measured)	m/z (calculated)	Error / ppm
$[Sulfatide-H]^-$	888.625	888.62346	1.7330
$[PG(34:1)+H]^+$	749.531	749.53326	3.0152
$[PG(34:1)+Na]^+$	771.513	771.51520	2.8515
$[PG(34:1)+K]^+$	787.487	787.48914	2.7175
$[PE(34:1)+H]^+$	718.538	718.53868	0.9464
$[PE(34:1)+Na]^+$	740.520	740.52062	0.8372
$[PE(34:1)+K]^+$	756.494	756.49456	0.7403
$[PE(34:1)-H]^-$	716.524	716.52303	1.3538
$[PC(34:1)+H]^+$	760.585	760.58563	0.8283
$[PC(34:1)+Na]^+$	782.567	782.56757	0.7284
$[PC(34:1)+K]^+$	798.541	798.54151	0.6387
$[PC(38:4)+H]^+$	810.598	810.60128	4.0464
$[PC(38:4)+Na]^+$	832.583	832.58322	0.2642
$[PC(38:4)+K]^+$	848.556	848.55716	1.3670
$[PC(40:6)+H]^+$	834.600	834.60128	1.5337
$[PC(40:6)+Na]^+$	856.582	856.58322	1.4243
$[PC(40:6)+K]^+$	872.556	872.55716	1.3294

Table S4 Average signal intensity of different lipids in white matter and molecular layer as determined by MALDI- and MALDI-2- MSI in positive ion mode with DHAP as a matrix

	white matter		molecular layer	
	MALDI	MALDI-2	MALDI	MALDI-2
PG(34:1)	1.13E+02	2.33E+02	1.72E+02	1.77E+03
PE(34:1)	7.10E+01	1.52E+04	6.58E+01	2.39E+04
PC(34:1)	1.89E+04	1.43E+04	2.34E+04	4.95E+04
PC(38:4)	1.72E+03	9.94E+02	2.05E+03	4.24E+03
PC(40:6)	2.03E+03	1.31E+03	5.29E+03	1.02E+04

Table S5 Average signal intensity of different lipids in white matter and molecular layer as determined by MALDI- and MALDI-2- MSI in negative ion mode with DHAP as a matrix. Sulfatide: (3'-sulfo)Galβ1-1Cer(d18:1/24:1)

	white matter		molecular layer	
	MALDI	MALDI-2	MALDI	MALDI-2
Sulfatide	3.38E+04	1.16E+03	1.02E+03	1.17E+01
PG(34:1)	1.56E+02	4.69E+02	5.05E+02	1.42E+03
PE(34:1)	1.49E+02	1.25E+03	1.58E+02	9.45E+02
